# Ionospheric Phase Compensation for InSAR Measurements Based on the Faraday Rotation Inversion Method

**DOI:** 10.3390/s20236877

**Published:** 2020-12-01

**Authors:** Bing Li, Zemin Wang, Jiachun An, Baojun Zhang, Hong Geng, Yuanyuan Ma, Mingci Li, Yide Qian

**Affiliations:** 1 Chinese Antarctic Center of Surveying and Mapping, Wuhan University, 129 Luoyu Road, Wuhan 430079, China; binglee@whu.edu.cn (B.L.); zmwang@whu.edu.cn (Z.W.); bjzhang@whu.edu.cn (B.Z.); mayuanyuan123@whu.edu.cn (Y.M.); mingcich@163.com (M.L.); qianyide@whu.edu.cn (Y.Q.); 2 State Key Laboratory of Information Engineering in Surveying, Mapping and Remote Sensing, Wuhan University, Wuhan 430079, China; 3 School of Resource and Environmental Sciences, Wuhan University, Wuhan 430079, China; genghong@whu.edu.cn

**Keywords:** radar interferometry, ionospheric distortion, Faraday rotation, compensation

## Abstract

The ionospheric error can significantly affect the synthetic aperture radar (SAR) signals, particularly in the case of L band and lower frequency SAR systems. The ionospheric distortions are mixed with terrain and ground deformation signals, lowering the precision of the interferometric measurements. Moreover, it is often difficult to detect the small-scale ionospheric structure due to its rapid changes and may have more influence on ionospheric phase compensation for InSAR measurements. In this paper, we present a Faraday rotation (FR) inversion method and corresponding procedure to compensate the ionospheric error for SAR interferograms and to detect the variations of small-scale ionospheric disturbances. This method retrieves the absolute total electron content (TEC) based on the FR estimation and corrects the ionospheric error for synthetic aperture radar interferometry (InSAR) measurements by transforming the differential TEC into the ionospheric phase. In two selected study cases, located in high latitude and equatorial regions where ionospheric disturbances occur frequently, we test the method using the Phased Array L-band Synthetic Aperture Radar (PALSAR) full-polarimetric SAR images. Our results show that the proposed procedure can effectively compensate the ionospheric phase. In order to validate the results, we present the results of ionospheric phase compensation based on the split-spectrum method as a comparison to the proposed method. To analyze the ability of our proposed method in detecting small-scale ionospheric disturbances, TEC derived from FR estimation are also compared with those derived from the global ionosphere maps (GIM). Our research provides a robust choice for the correction of ionospheric error in SAR interferograms. It also provides a powerful tool to measure small-scale ionospheric structure.

## 1. Introduction

Synthetic aperture radar interferometry (InSAR) technology has been widely used to measure Earth’s topography and to study geophysical phenomena, such as earthquakes, volcanoes, city subsidence, landslides, and glacier movements [1]. The accuracy of InSAR measurements can be affected by various noise sources, including orbital error, atmospheric error, residual topography error, and decorrelation noise. However, recent studies show that ionospheric error, which was often neglected before, can significantly affect the synthetic aperture radar (SAR) signals, particularly in the case of L band and lower frequency SAR systems [2,3,4,5,6]. The ionospheric distortions are embodied by phase advance, phase or amplitude scintillation, and Faraday rotation (FR) in the signal polarization [7,8,9]. In SAR interferograms, the ionospheric distortions mixed with the topography and ground deformation phase is usually mistaken for troposphere or orbit error and removed by polynomial fitting with uncertain accuracy. The estimation and compensation of the ionospheric phase are therefore necessary in order to separate the ionospheric phase error from interferograms and improve the InSAR measurement accuracy.

Recently, several approaches for the ionospheric phase compensation of SAR Interferograms have been proposed, including the azimuth shift method, the range group-phase delay difference method, the range split-spectrum method, and the FR inversion method [10]. The azimuth shift method utilizes the proportional relation between the azimuth gradient of ionospheric phase delay and the azimuth displacement [11,12,13], and can be estimated from azimuth offset tracking [14] and multiple-aperture interferometry (MAI) [15]. This method requires that the azimuth displacement caused by ground deformation can be overlooked, and cannot recover the ionospheric variations along the range direction. The range phase-group delay difference method is based on the fact that the ionospheric phase and group delays are equal in magnitude, but opposite in sign [16,17]. The range split-spectrum technique uses the dispersive characteristic of the ionosphere and divides the range spectrum of the radar signal into two sub-bands to estimate the ionospheric phase component [18,19,20,21,22,23]. An 80 MHz bandwidth seems enough for the needed separation of the sub-bands [24]. However, these two methods are prone to constrain by the range bandwidth of existing SAR systems, and the derived initial ionospheric phase is quite noisy. Furthermore, in areas with low coherence, the phase unwrapping error may become a problem for the compensation methods in which involve this operation.

FR inversion method is a new technique to image the ionosphere and shows great promise for the ionospheric correction [24,25]. Compared with ionosphere inversion by Global Navigation Satellite System (GNSS) techniques, FR method can realize the imaging of small-scale ionospheric structure, such as aurora-associated ionospheric enhancement and plasma bubbles [25]. The absolute total electron content (TEC) can be derived from FR estimation [26]. By differencing the derived TEC information, the differential ionospheric phase is calculated to compensate the ionospheric distortions in the SAR interferograms. However, as the only correction method that can retrieve the absolute ionospheric phase, the FR inversion method has not received enough attention. The related research is still limited [27,28]. 

In this paper, we present a FR inversion method and operational procedure to compensate the ionospheric phase for SAR interferograms. In Section 2 of this paper, we present the derivation of the FR inversion method. Section 3 introduces the datasets and study area. An overall scheme of the implementation of the method is presented, and its key points are analyzed in detail. Section 4 presents the experimental results of the ionospheric phase compensation based on the proposed procedure. In Section 5, the ionospheric phase compensation based on the split-spectrum method was selected as a comparison. We also use the global ionosphere maps (GIM) model to validate our results. In Section 6, the findings are summarized, and future research work is proposed.

## 2. Methods

### 2.1. Ionosphere Effects on Radar Interferometry

The high energy EUV, and X-ray photons from the sun lead to the ionization of the atmosphere, leaving the ionosphere in a mixture of free electrons, ions, and neutral gases [29]. The density of free electrons Ne is closely related to the sun’s activity, atmospheric density profile, geographic location, magnitude and orientation of the Earth’s magnetic field, and time of day [30]. The ionosphere suffers disturbances during geomagnetic storms which are called ionospheric storms. The disturbed ionosphere is characterized as a large increase or depletion of electron density from their normal level. As one of the fundamental equations, the formula of the ionospheric refractive index, often referred to as the Appleton-Hartree formula [31], is used to describe ionospheric effects on the electromagnetic wave signal. Two main effects can be derived from the Appelton-Hartree equation: Phase advance and FR. For SAR interferograms, the distorted ionospheric phase can be expressed as

(1) $$\varphi_{iono}=-\frac{4\pi K}{cf_{0}}\mathrm{TEC}$$

where *K* is a constant 40.28 m^3^/s^2^, *f*_0_ is the radar center frequency, and *c* is the speed of light in the vacuum. The slant TEC can be calculated by integrating Ne between the satellite and the target, along a tube of 1 m^2^ cross-section. The negative sign in (1) denotes that the ionospheric contribution is a phase advance.

### 2.2. Derivation of the FR Inversion Method 

Besides phase advance, another ionosphere effect on SAR interferograms is FR, which rotates SAR polarizations during their two-way transmission through the ionosphere. Although FR has little effect on interferometry measurements, via FR inversion, TEC can be derived. Just based on this point, our study achieves ionospheric phase compensation for InSAR measurements.

#### 2.2.1. FR Calculation Based on Appleton-Hartree Formula

FR is closely related to the Earth’s magnetic field, the ionospheric TEC, and the geometry of the observation by the following Equation [25]

(2) $$\Omega_{AH}=\frac{2.365\;\times \;10^{4}}{{f_{0}}^{2}}B\cos\psi \mathrm{TEC}$$

where *B* is the intensity of the Earth’s total magnetic field at 350 km altitude, and *ψ* are the angles between the vectors of radio wave and the Earth’s magnetic field. *Ψ* is calculated by

(3) cosψ=cosθsinα+sinθcosαsinβ

With the off-nadir angle of the SAR sensor *θ*, the magnetic inclination angle α, and the magnetic declination angle *β.*

#### 2.2.2. Estimation of FR Angle from Full-polarimetric SAR Images

The presence of nonzero FR means that cross-polarization measurements will not be reciprocal [32]. Suppose that the FR angle is the only error source, the FR angle can be estimated from the measured scattering matrix. At present, there are two types of FR estimation methods. The first is based on scattering matrix data, such as proposed in [33]. The second exploited the elements of covariance matrix data or polarimetric coherency matrix [32,34,35,36]. In our study, an FR estimator based on the polarimetric coherency matrix is adopted and given by [37]

(4) ΩT=14Arg{(T11−T44)−2jIm(T14)}

where, *T_11_*, *T*_14_, *T_44_* are the elements of 4 × 4 polarimetric coherency matrix, *T*_4_ = <*U*_p_*U*_p_^H^>. j denotes an imaginary number unit, *j*^2^ = −1. <·> indicates temporal or spatial ensemble averaging, assuming homogeneity of the random medium. ’H’ represents matrix conjugate transposition operations. *U*_p_ is the 4-dim Pauli feature vector [38]. 

#### 2.2.3. Ionospheric Phase Calculation

Once FR is calculated from Equation (3), via Equation (2), TEC can be obtained by the following Equation

(5) $$\mathrm{TEC}=\frac{{f_{0}}^{2}\Omega_{T}}{2.365\times \;10^{4}B\cos\psi }$$

Subsequently, according to Equation (1), the differential ionospheric phase in SAR interferograms from *t*_1_ to *t*_2_ is given by

(6) $$\Delta \varphi_{iono}=-\frac{4\pi Kf_{0}\Delta {\Omega_{T}}^{t_{1}t_{2}}}{2.365\times \;10^{4}cB\cos\psi }$$

#### 2.2.4. Accuracy of the FR-TEC Inversion Method 

Based on the above deduction, it can be found that the accuracy of FR inversion method mainly depends on the estimated FR angle Ω_T_ and the Earth’s total magnetic field B. In our study, the 13th generation international geomagnetic reference field (IGRF) was adopted to estimate the magnetic field at the accuracy about 50–300 nT [39]. Fluctuations in the magnetic field caused by ionospheric and magnetospheric disturb are usually less than 1%, or a few percent under severe magnetic storms compared with the intensity of the total magnetic field. The accuracy of IGRF has been validated by in-situ satellites with magnetometers, and the discrepancy between in-situ satellite data and the IGRF-10 at lower altitudes in the polar region has been substantially improved in the IGRF-11 [40]. Therefore, FR becomes the main error source in the FR inversion method in this study. The ionospheric phase is proportional to the FR; from Equation (5) we can write

(7) $$\sigma_{\Delta \varphi_{iono}}^{2}=\frac{4\pi Kf_{0}}{2.365\times \;10^{4}cB\cos\psi }\sigma_{\Delta \Omega_{T}^{t_{1}t_{2}}}^{2}$$

## 3. Materials and Implementation

### 3.1. Datasets and Study Area

The L-band Advanced Land Observation Satellite (ALOS) is an Earth science mission launched by the Japan Aerospace Exploration Agency (JAXA) on 24 January 2006, orbiting at about 691 km altitude [41]. The Phased Array L-band Synthetic Aperture Radar (PALSAR) operated on board the ALOS platform to achieve cloud-free and day-and-night land observation. In our study, in order to assess the performance of the ionospheric correction of the FR inversion method, we selected two representative experimental areas where ionospheric disturbances occur frequently. Details of information about these two interferometric pairs are shown in Table 1. 

**Figure 1:**
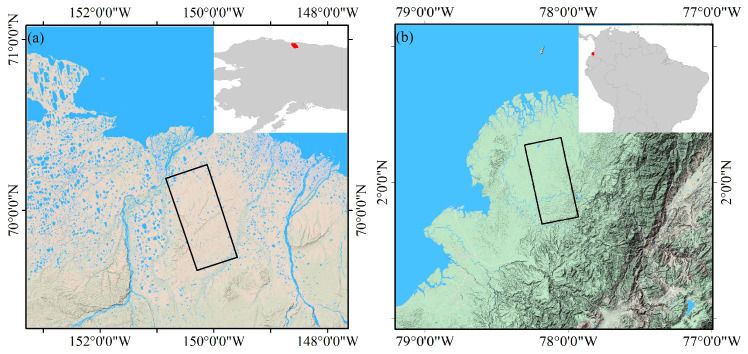
The coverage and map projection of PALSAR images to be presented in our study for ionospheric phase compensation at (**a**) Alaska and (**b**) Equator.

Figure 1 illustrates the coverage and map projection of the PALSAR images. We chose two representative experimental areas. Two case studies are located in high latitude and equatorial regions where ionospheric disturbances occur frequently. At the geomagnetic equator, the variations of the magnetic field in tens to hundreds of nT can occur, while in the auroral region at high latitudes, it can be hundreds to thousands of nT. The first example is the ionospheric phase correction under the condition of auroral activity in northern Alaska, which is located in the aurora zone. In this interferometric pair, the master image acquired at the time of the ALOS pass has been confirmed to be disturbed by significant ionospheric activity, and the geomagnetic variations achieved almost 1000 nT during the disturbed day on 1 April 2007. Checking the space weather and geophysical conditions, we learned that a solar wind stream hit the Earth, causing visible auroras all around the North Polar Region [42]. The other case is the compensation under the equatorial ionospheric disturbance. The interferometric pair is located near the equator, where ionospheric anomaly and scintillation occur frequently.

**Figure 2:**
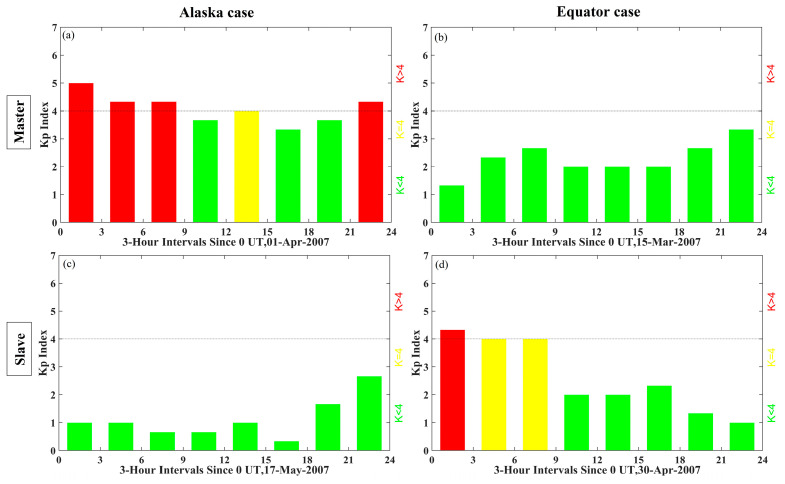
(**a**,**c**) the global geomagnetic activity index Kp corresponds to the Alaska case, (**b**,**d**) the index Kp corresponds to the Equator case. The red, yellow and green squares indicate different levels of geomagnetic activities.

The global geomagnetic activity Kp-index was used to characterize the ionospheric response at the SAR imaging time. The name Kp has a German origin and is an acronym for “planetarische Kennziffer,” which simply means planetary index [43]. The values of the Kp range from 0 (very quiet) to 9 (very disturbed), and generally it is considered that a strong geomagnetic disturbance occurs when Kp exceeds 4. In the Alaska case, the Kp index (Figure 2a) shows that a strong geomagnetic activity occurred during the master acquisition (UT 07:29:39, 1 April 2007), whereas the Kp index in the Equator case (Figure 2d) indicates that the geomagnetic disturbance happened during the slave acquisition (UT 03:51:10, 30 April 2007).

### 3.2. Experiment Implementation

#### 3.2.1. Data Processing for the FR Inversion Method

**Figure 3:**
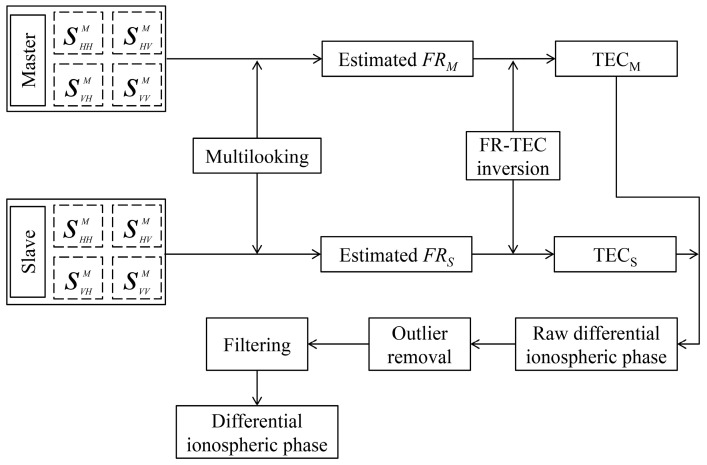
Schematic representation of workflow.

As shown in Figure 3, the process flow of our proposed approach is summarized. First, the calibration should be carefully performed for the qualitative use of SAR data. Then, construct the polarization coherence matrix according to the calibrated polarization scattering matrix, followed by a multi-looking procedure for noise reduction. After that, the FR estimates (*FR_M_*, *FR_S_*) of master and slave images are computed according to Equation (4), respectively.

In the second step, the corresponding TEC images (TEC_M_, TEC_S_) of each interferometric pair are derived from the FR estimates. Afterwards, the raw ionospheric phase was estimated via Equation (6). For those pixels without backscatter information (such as water body and shadows), outlier removal is necessary. In our experiment, those pixels whose phase values are larger than three times the root mean square error will be masked. A 2-D Gaussian weighted filter is adopted to smooth the estimated ionospheric phase [20]. The size of the filter window depends on the noise level. Finally, we get the differential ionospheric phase estimates that meet the requirement of ionospheric phase compensation.

#### 3.2.2. Ionospheric Phase Compensation for SAR Interferograms

The ionospheric error within the interferograms are compensated in this step. The InSAR phase consists of different phase components related to surface topography, surface deformation, atmospheric delay, the Earth curvature, as well as ionospheric effects. For InSAR measurements, the deformation phase can be expressed as follows [44]

(8) $$\varphi_{def}=\varphi_{InSAR}-(\varphi_{flat}+\varphi_{topo}+\varphi_{atm}+\varphi_{orbit})-\Delta \varphi_{iono}$$

where *φ_def_* is the surface deformation, *φ_InSAR_* is interferometric phase, *φ_flat_* represents the flat-Earth phase, *φ_topo_* denotes the topographic phase, *φ_atm_* indicates atmospheric delay, *φ_oribit_* is the orbital error, and Δ*φ_iono_* stands for the ionospheric phase. In this paper, we assumed that the surface deformation is negligible during the revisit period of the SAR sensor. In addition, our study did not consider atmospheric delay correction.

The topography-related phase is typically corrected using a Digital Elevation Model (DEM). Experience has shown that available baseline information, especially the one calculated from orbital data, may not be accurate enough, resulting in the occurrence of orbital error [45]. A polynomial fitting method, which is commonly adopted in the InSAR processing, was used to remove the phase distortion induced by the orbital error.

## 4. Experimental Results 

### 4.1. FR Estimation 

**Figure 4:**
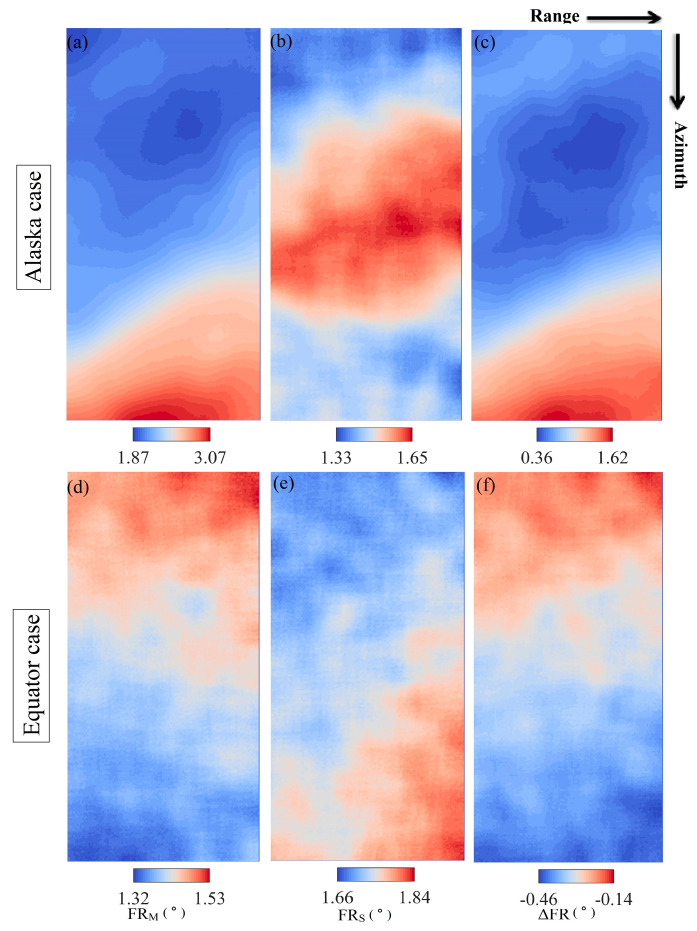
(**a**–**c**) Estimated Faraday rotation (FR) for the master and slave, and differential FR in the Alaska case. (**d**–**f**) Estimated FR and differential FR in the Equator case.

FR estimation is a key step in the process of the FR inversion. Before the calculation, the SAR images are processed by a multi-looking operation of 7 pixels in the azimuth and 1 pixel in the range directions to reduce speckle noise. Utilizing the elements in the polarization coherence matrix, the FR values can be calculated by Equation (4), and followed by a 2-D Gaussian weighted filter with a window size of 128 on the FR images. Estimated FR for the master and slave images and the differential FR images are presented in Figure 4.

### 4.2. Magnetic Field Calculation

**Figure 5:**
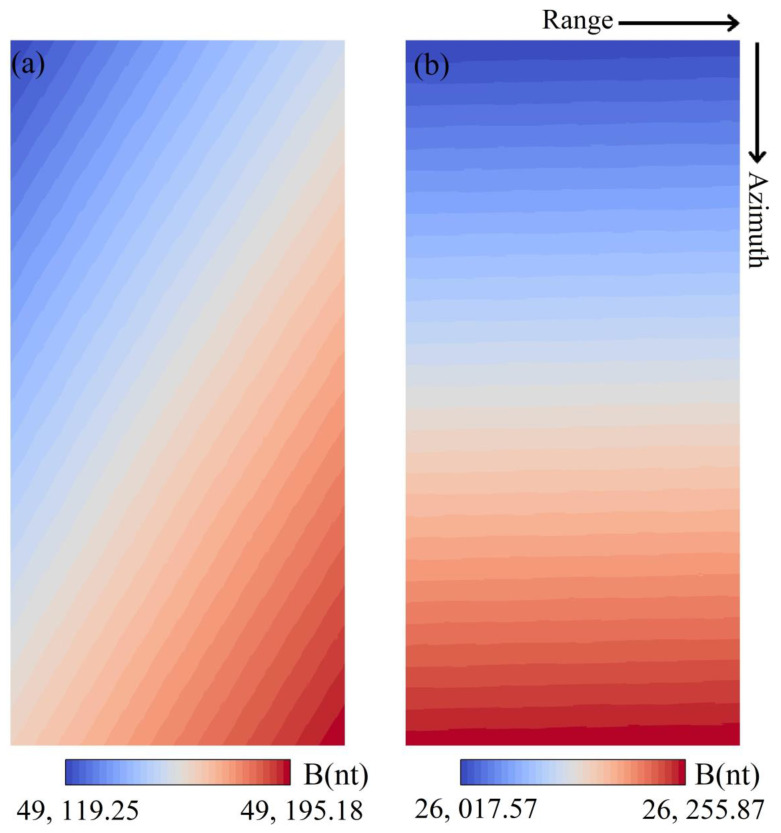
The intensity of the Earth’s magnetic field for two interferometric pairs: (**a**) The Alaska case, (**b**) the Equator case.

The intensity of the Earth’s magnetic field is also an essential element for calculating the ionosphere phase. In our study, considering the small inter-annual variation of the magnetic field, the magnetic field strength at the master image acquisition was selected as the input parameter. The geomagnetic field was collected from geomagnetic data provided by the National Geophysical Data Center (https://www.ngdc.noaa.gov/). To improve the computational efficiency, calculations were first generated at 0.1° increments in latitude and longitude covering the experimental area, followed by Kriging interpolation and coordinate conversion. After this operation, the magnetic field images were obtained in the SAR geometry (Figure 5).

### 4.3. Ionospheric Phase Compensation

**Figure 6:**
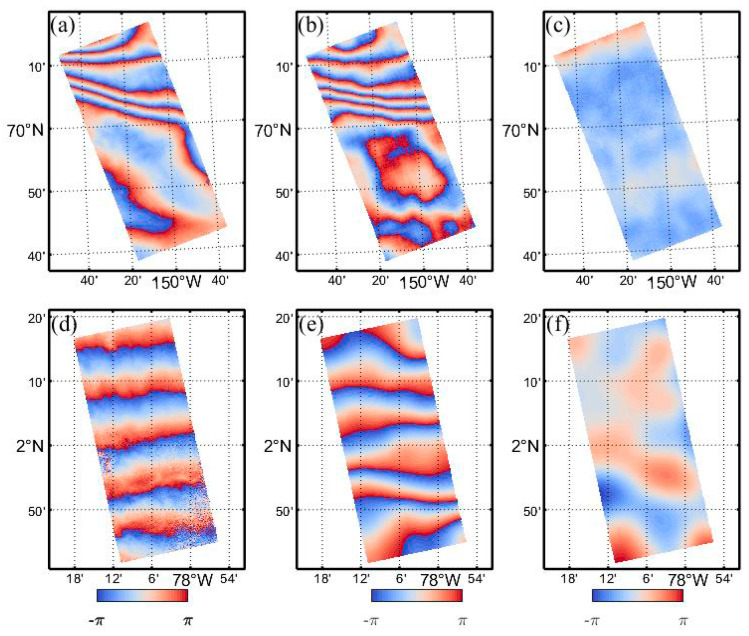
Ionospheric compensation: (**a**,**d**) interferograms without ionospheric correction, (**b**,**e**) estimated ionosphere phase screen (wrapped) derived from the algorithm described in our study, (**c**,**f**) interferograms with ionospheric compensation.

Based on the HH-polarization SAR data, the topographic phase-removed differential interferograms were generated and presented in Figure 6a,d, in which the ionospheric disturbances are clearly visible. Using the proposed procedure in Section 3.2, we extracted the differential ionospheric phase between two SAR acquisitions, and the results are presented in Figure 6b,e.

As shown in Figure 6, fringe patterns of the ionospheric phase are consistent with the differential interferograms. The ionospheric phase in the Alaska case distributes irregularly: The upper half of the image fluctuates more rapidly than the lower part, while in the equatorial case, the fringes are regularly arranged along the azimuth direction, which can be easily mistaken as orbit errors if not carefully identified. Since the ionospheric correction cannot compensate for the longwave signal related to orbit error, a separate baseline fitting is still necessary after the initial ionospheric correction step. According to Equation (8), the topographic phase, ionospheric phase, and baseline error-corrected interferograms are generated and displayed in Figure 6c,f.

By the visual inspection, all strong phase ramps caused by the ionosphere disturbance have been successfully removed. However, there remains a residual phase in the ionosphere-compensated interferograms, which are mainly composed of the data processing error, tropospheric error, magnetic field model error, and residual ionospheric error. The remaining tropospheric error could be mitigated by using numerical weather models or other external data. The data processing error mainly causes by filtering operations, which may blur the high-frequency components of the ionosphere signals. The IGRF model has a lower accuracy under severe ionospheric disturbances, which may also affect the accuracy of the FR inversion method. The quantitative analysis is presented in Section 5.

## 5. Validation and Discussion

### 5.1. Comparison with the Split-Spectrum Method

In order to validate the proposed method, another method that has been put forward to compensate for the ionospheric phase, the split-spectrum method, was used in the comparative experiment. The split-spectrum method exploits the dispersive nature of ionospheric to compensate ionospheric phase. Taking this basic concept, the range spectrum of the radar signal is divided into two sub-bands with equal bandwidth implemented by applying a band-pass filter. Equation (1) shows that the ionospheric phase is inversely related to the center frequency *f*_0_. The interferometric phase can be decomposed into a depressive component and non-dispersive component resulting in a simplified form [18]

(9) $$\Delta \varphi_{L}=\Delta \varphi_{non-disp}\frac{f_{L}}{f_{0}}+\Delta \varphi_{iono}\frac{f_{0}}{f_{L}}$$

(10) $$\Delta \varphi_{H}=\Delta \varphi_{non-disp}\frac{f_{H}}{f_{0}}+\Delta \varphi_{iono}\frac{f_{0}}{f_{H}}$$

where Δ*φ_L_* and Δ*φ_H_* stand for the interferometric phase corresponding to sub-bands with center frequencies of *f_L_*, *f_H_*, respectively. By solving the linear system, the dispersive Δ*φ_iono_* component can be estimated as follows

(11) $$\Delta \varphi_{iono}=\frac{f_{H}f_{L}}{f_{0}(f_{H}^{2}-f_{L}^{2})}(\Delta \varphi_{L}f_{H}+\Delta \varphi_{H}f_{L})$$

**Figure 7:**
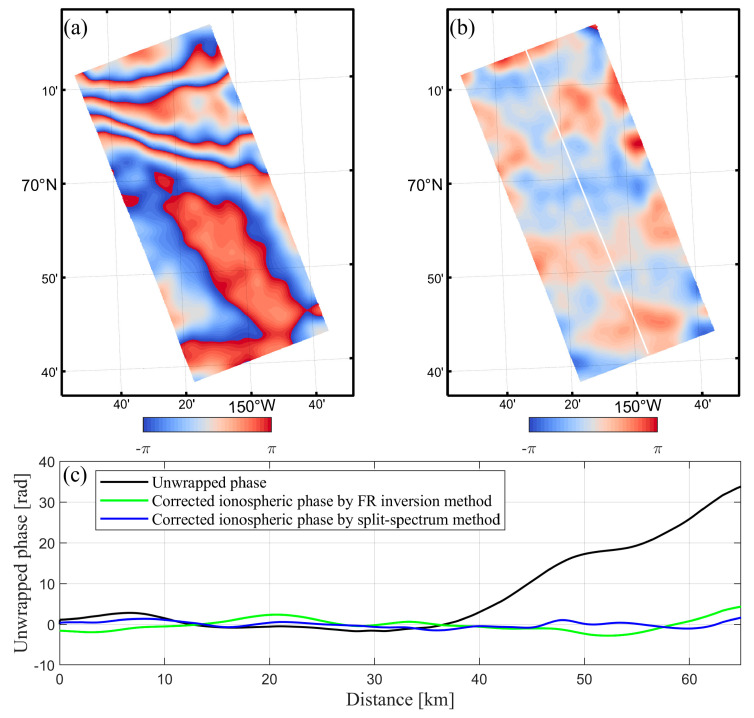
Ionosphere phase compensation of the Alaska case derived from the split-spectrum method: (**a**) The estimated ionospheric phase screen, and (**b**) ionosphere-compensated interferogram, (**c**) phase comparison with and without ionosphere correction along the profile in white line.

**Figure 8:**
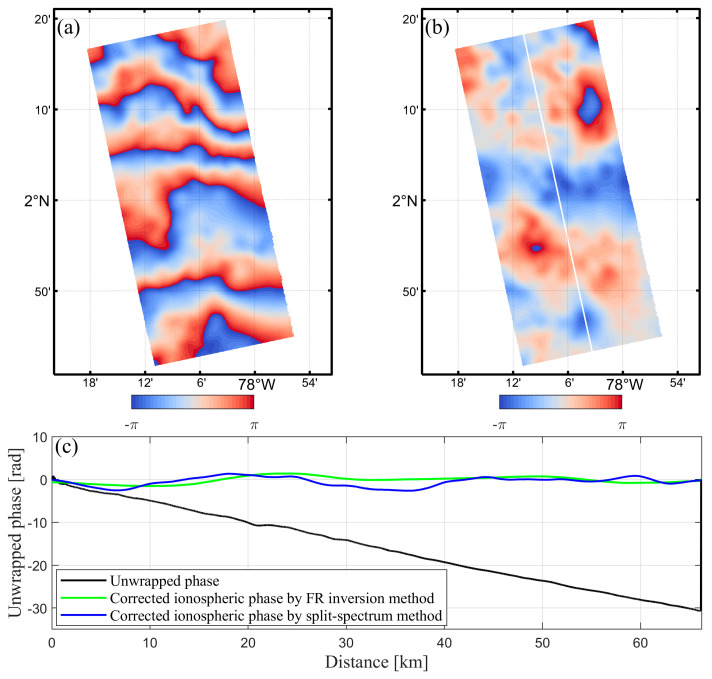
Ionosphere phase compensation of the Equator case derived from the split-spectrum method: (**a**) The estimated ionospheric phase screen, and (**b**) ionosphere-compensated interferogram, (**c**) phase comparison with and without ionosphere correction along the profile in white line.

We calculated the ionosphere phase and ionosphere-compensated interferograms based on the split-spectrum method and carried out phase unwrapping using the minimum cost flow (MCF) method. The ionospheric phase estimation and compensation for the Alaska case (Figure 7a,b) and the Equator case (Figure 8a,b) are shown in Figure 7 and Figure 8, respectively. 

To provide a quantitative analysis, the two ionospheric correction methods were compared below. As illustrated in Figure 7c, in the Alaska case, without ionospheric error correction, the maximum phase values along the profile in the original interferogram reach up to 33.8 rad, which means the corresponding measurement error can reach to 0.64 m in the line-of-sight (LOS) direction. By performing the ionospheric phase compensation based on the FR inversion method and the split-spectrum method, the phase mean values of the interferograms reduced to −0.19 and −0.04 rad, respectively. It should be noted that the accuracy of the IGRF model may degrade in the polar region. This might be related to the strong distortion of the geomagnetic field by the intense auroral activity. In the Alaska case, the geomagnetic variations at high latitudes achieved almost 1000 nT during the disturbed day on 1 April 2007. This may be the reason that the correction performance of the split-spectrum method is better than that of the FR inversion method.

In the Equator case (Figure 8), the maximum value along the interferogram phase curve can reach up to −30.7 rad, with a measurement error of −0.58 m. The mean phase value of the original interferograms decreased to −0.12 and −0.54 rad, respectively, by applying the FR inversion method and the split-spectrum method. For the whole interferogram images, the mean phase values of the interferograms drop from 7.2 and −15.5 rad to near zero after ionosphere phase compensation, indicating that the ionosphere effect is the main error source in our study. The statistical results show that the FR inversion, as well as the split spectrum method, both can effectively correct the ionospheric phase error in the interferograms.

The fringe patterns of the estimated ionospheric appear to be noisier and more irregular compared with those derived by the FR inversion method. Furthermore, obvious residual long-wavelength signals remain in the original interferograms after ionosphere compensation, which are not observed in the FR inversion method. Since the final estimation accuracy of the split-spectrum method depends on the carrier frequency and bandwidth, this can greatly affect the experimental results in view of the narrow range bandwidth (14 MHz) of our SAR data. In addition, the success of the split-spectrum method also involves several factors in interferometric processing, such as coherence related to scene characteristics and phase unwrapping. In contrast to other ionospheric correction methods, the most significant advantage of the FR inversion method is that the interferometric errors, such as the interferometric coherence and unwrapping error, have no effect on the estimation accuracy of ionospheric phase. In fact, the FR inversion method is mainly affected by the scene characteristics, and therefore robust enough compared with the split-spectrum method.

### 5.2. Comparison with TEC Maps

In this section, estimated TEC is compared with that obtained from external observation. As a commonly used technique, GNSS observation has been widely used in monitoring, tracking, and forecasting ionospheric perturbations. Given that there were few GNSS stations available in our experimental area, we chose the GIM as a reference and compared differential TEC results. GIM are generated on a daily basis by different ionospheric associate analysis centers with a temporal resolution of 2 h [46]. The maps need to be interpolated in both time and space to obtain the TEC estimation for the ionospheric piercing point corresponding to each pixel of SAR image. Since the calculation results of the GIM TEC maps are a vertical TEC (VTEC), differential VTEC derived from the FR inversion should be converted to differential TEC (dTEC) via

(12) $$\Delta \mathrm{TEC}=({\mathrm{TEC}}_{m}-{\mathrm{TEC}}_{s})\frac{1}{\cos\theta }$$

where TEC*_m_* and TEC*_s_* are, respectively, the VTEC during master and slave acquisitions.

**Figure 9:**
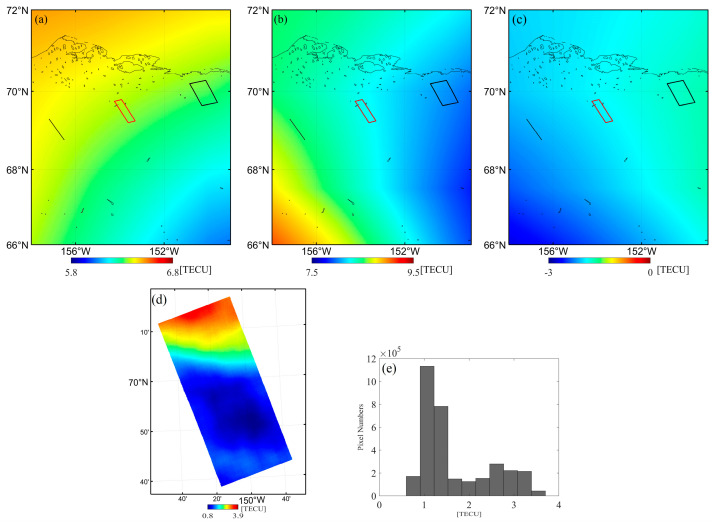
Total electron content (TEC) comparison in the Alaska case: (**a**,**b**) TEC maps derived from the GIM model for master and slave acquisition, respectively. The black line and square represent the orbit and image ground footprint; the red square indicates the crossed region projected to the ionosphere; (**c**,**d**) differential TEC obtained by the GIM model and FR inversion, respectively. (**e**) The histogram of (**d**).

In the Alaska case, Figure 9a,b shows the TEC maps derived from the GIM model for master and slave, respectively. Assuming a single-layer model at the height of 350 km, the red square in Figure 9 depicts the ionospheric pierce region that SAR signal project on the thin layer. The differential ionosphere was compared under this thin layer. Results show that computed by the GIM model (Figure 9c) has only a single value of −1.9 TECU within the ionospheric pierce region, while the corresponding dTEC from the FR inversion (Figure 9d) range from 0.8 to 3.9 TECU. We also counted the pixels of the whole FR-inversion dTEC image. The statistics indicate that the dTEC values between [0.9, 1.5] displayed in blue color in Figure 9e account for the largest proportion.

**Figure 10:**
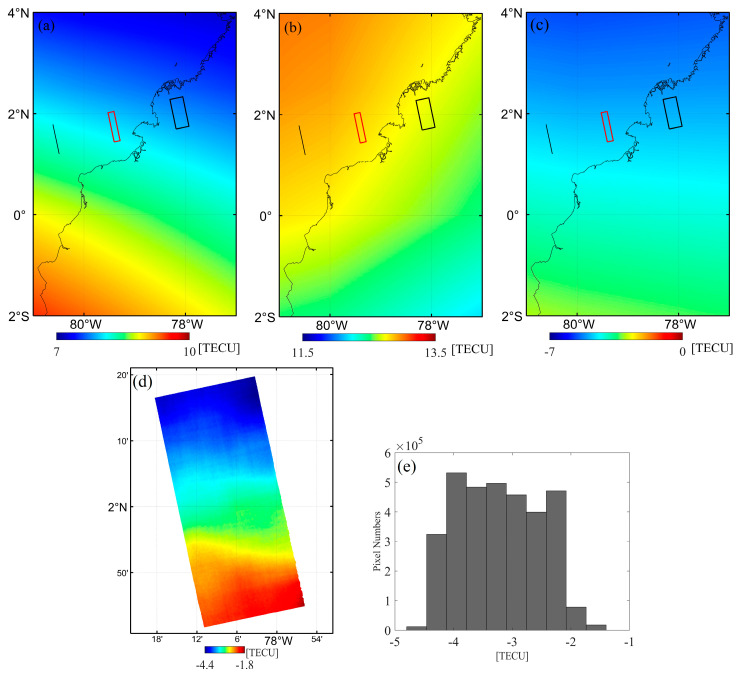
TEC Comparison in the Equator case: (**a**,**b**) TEC maps derived from the GIM model for master and slave acquisition, respectively. The black line and square denote the orbit and image ground footprint; The red square indicates the crossed region projected to the ionosphere; (**c**,**d**) differential TEC obtained by the GIM model and FR inversion, respectively. (**e**) The histogram of (**d**).

In the Equator case, TEC maps for the master and slave derived from the GIM model are displayed in Figure 10a,b, respectively. The dTEC values derived from the GIM model (Figure 10c) within the ionospheric pierce region vary from −4.9 to −4.6 TECU, with an average value of −4.7 TECU, while the corresponding dTEC from the FR inversion (Figure 10d) range from −4.4 to −1.8 TECU, with an average value of −3.2 TECU. The histogram in Figure 10e indicates that the dTEC are evenly distributed within the image. Besides, we find that the dTEC maps in Figure 10c,d both have an ionospheric gradient along the NW–SE direction.

The results above show that the GIM model can basically describe the TEC distribution derived by the FR inversion method. The FR-TEC has obvious advantages over GIM-TEC in the detection of small-scale ionosphere. Using a regional ionospheric model, it is possible to get a better estimate of the real ionosphere conditions and then apply to the ionospheric phase compensation. However, it still needs to be verified by specific experiments.

## 6. Conclusions

In this paper, we present a FR inversion method and establish an operational procedure for compensation of ionospheric effects in SAR interferograms. Two case studies located in high latitude and equatorial regions where ionospheric disturbances occur frequently were used to demonstrate the performance of the proposed FR inversion method. The results show that the proposed method can effectively compensate for the ionospheric phase error in InSAR measurements.

We compared the proposed method with another ionosphere correction method, namely the split-spectrum method, and carried out a quantitative analysis. The results show that both the method can effectively correct the ionospheric phase error in the interferograms, but the ionospheric phase derived from the split-spectrum method shows noisier and more irregularity. After applying both ionosphere correction methods, the phase values of the interferograms decreased to near zero, which means that the ionosphere effect is the dominant error in our study. In contrast to the split-spectrum method, the implementation of our proposed method is affected by fewer error sources, and therefore more robust. The estimated ionosphere was also validated with the TEC maps derived from the GIM. The results show that the GIM can generally characterize the spatial variation of the ionosphere derived by the FR inversion method. 

To sum up, the FR inversion method can not only be used for ionospheric correction in InSAR, but more importantly, it provides an effective ionospheric detection method. Especially in the detection of small-scale ionospheric anomalies, the FR inversion method can extract ionospheric anomalies with extremely high spatial resolution, far better than GNSS-TEC. In terms of SAR-based ionospheric inversion, the FR inversion method can extract the absolute variations of ionosphere, while the split-spectrum method can only obtain the relative variations. Furthermore, the proposed method does not affect by the phase unwrapping, which may become a problem for other compensation methods (e.g., the azimuth shift method, the split-spectrum method) involving this operation.

It should be noted that full-polarimetric SAR images are needed in our proposed method. However, this unsatisfactory situation has gradually changed in the last decade with the launch of a number of SAR satellites operating with polarimetric capability at different frequencies, e.g., ALOS-1, ALOS-2, RADARSAT-2, Gaofen-3, and also future planned missions (e.g., ALOS-4, Tandem-L). Besides, inappropriate filtering may blur the high-frequency components of the ionosphere signals. A better filtering strategy, which should reduce the noise variance while respecting high-frequency ionospheric signals, is in need to improve the results. In view of the remarkable changes in the magnetic field, when the ionosphere disturbances happen, this might also affect the accuracy of the FR inversion method. Our future research will focus on the influence of different geomagnetic models. Another possibility, to make the compensation results more precise, is the combination of the multi-ionospheric phase compensation methods, such as the split-spectrum method, MAI-based ionosphere correction method, and the proposed FR inversion method.

## Figures and Tables

**Table 1:** Dataset information of application examples.

Experiment Region	Track No.	Frame	Master	Slave
Alaska dataset	243	1410	1 April 2007	17 May 2007
Equator dataset	150	0030	15 March 2007	30 April 2007
